# Preventive Effect of Powder Bergamot Juice (C*itrus bergamia* Risso & Poiteau) on Pathophysiological Processes of Renal Disease in an Experimental Western Diet Model

**DOI:** 10.1002/mnfr.70411

**Published:** 2026-02-09

**Authors:** Marina de Paula Salomé dos Santos, Juliana Silva Siqueira, Erika Tiemi Nakandakare‐Maia, Taynara Aparecida Vieira, Núbia Alves Grandini, Thiago Luiz Novaga Palacio, Luís Eduardo Sormani, Caíque Manentti Caccia, Jordanna Cruzeiro, Fabiane Valentini Francisqueti‐Ferron, Camila Renata Corrêa

**Affiliations:** ^1^ Botucatu Medical School São Paulo State University (Unesp) Botucatu Brazil; ^2^ Department of Pharmaceutical Sciences University of Milan Milan Italy; ^3^ Integrated Colleges of Bauru (FIB) Bauru Brazil

**Keywords:** flavonoids, kidney disease, obesity, redox imbalance

## Abstract

The Western diet (WD), rich in sugars and saturated fats, is linked to metabolic disturbances that contribute to chronic kidney disease (CKD). Natural antioxidants have been investigated in several diseases, including bergamot (*Citrus bergamia*), a citrus fruit rich in polyphenols with recognized antioxidant and anti‐inflammatory properties. This study evaluated the preventive effect of powder bergamot juice (PBJ) on renal alterations induced by WD in male Wistar rats. Animals were allocated into four groups (*n* = 7): control diet, control + PBJ (250 mg/kg), WD; and WD + PBJ for 20 weeks. WD groups also received 25% sucrose in drinking water. Nutritional, metabolic, hormonal, renal, and oxidative stress (OS) markers were measured. Statistical analyses included two‐way ANOVA/Tukey's post‐hoc and Kruskal–Wallis/Dunn's post‐hoc (*p* < 0.05). WD increased caloric intake, body weight, adiposity index, glucose, triglycerides, and renal OS markers, and reduced kidney function. PBJ supplementation reduced adiposity and triglycerides, improved antioxidant enzyme activity, and preserved renal function. PBJ attenuated metabolic and oxidative alterations induced by WD, supporting its potential as a preventive strategy against early renal dysfunction associated with CKD.

## Graphical abstract

Excessive intake of sugars and fats contributes to obesity and renal dysfunction. Natural bioactive compounds, such as those found in bergamot (*Citrus bergamia*), have been studied for their protective potential. In an experimental rat model, bergamot supplementation reduced weight gain, improved kidney function, and decreased oxidative stress markers. Thus, bergamot may represent a preventive strategy against obesity‐related renal damage.

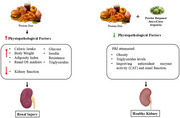

**Abbreviations**
4‐HNE: 4‐hydroxynonenal
8‐OHdG: 8‐hydroxy‐2’‐deoxyguanosine
AOPP: Advanced oxidation protein products
CAT: Catalase
CBO: Protein carbonylation
CKD: Chronic kidney disease
DNPH: 2,4‐dinitrophenylhydrazine
GFR: Glomerular filtration rate
HOMA‐IR: Homeostatic Model Assessment‐Insulin Resistance
IA: Adiposity index
LDL: Low‐density lipoprotein
MDA: Malondialdehyde
OS: Oxidative stress
PBJ: Powder Bergamot Juice
PBS: Phosphate‐Buffered Saline
ROS: Reactive oxygen species
SBP: Systolic blood pressure
SOD: Superoxid dismutase
TBA: Thiobarbituric acid
TyG: Triglyceride‐Glucose index
WD: Western diet

## 1 Introduction

The Western diet (WD) is a modern dietary pattern characterized by chronic consumption of foods rich in simple sugars and saturated fats [1]. The WD is associated with the development of obesity and complications such as dyslipidemia, hyperglycemia, arterial hypertension, and oxidative stress (OS) [2]. These conditions represent pathophysiological pillars in the development of chronic kidney disease (CKD) [3, 4, 5, 6, 7, 8, 9]. Therefore, preventive strategies for CKD are increasingly relevant in the context of modern dietary habits. [10, 11].

Natural antioxidant compounds have been investigated for their potential protective effects [12, 13, 14, 15, 16]. Bergamot (botanical name under the Vienna Code of 2006, *Citrus × bergamia* Risso & Poiteau [17] is mainly cultivated in a coastal geographical area of *Reggio* Calabria (Southern Italy) [18, 19] which includes three main cultivars: Castagnaro, Fantastico, and Femminello [20]. This citrus fruit is typically used in essential oils production, and the mixture of albedo, pulp, and seeds—known as *pastazzo*—represents the primary by‐product of this industrial process [15, 16, 19, 21]. Aiming at sustainable approaches, recent research has focused on this by‐product called powder bergamot juice (PBJ) [22, 23]. According to literature, bergamot fruit has demonstrated antioxidant, anti‐inflammatory, anti‐obesity, hypolipidemic, and hypoglycemic properties [15, 21, 24]. These effects are attributed to its high polyphenol content, including flavonoids such as naringin, neohesperidin, neoriocitrin, and melitidine [21].

Studies involving bergamot, kidney disease, and the WD remain limited. La Russa et al. reported that supplementation with the polyphenolic fraction of bergamot attenuated redox imbalance in obese rats, linking obesity‐related metabolic dysfunction to kidney injury [10]. In addition, treatments rich in polyphenols and flavonoids have been shown to reduce inflammation and OS, key factors involved in renal energy metabolism impairment induced by high sugar–fat diets [25].

Thus, the aim of this study was to evaluate the preventive effect of PBJ on pathophysiological processes involved in renal disease in rats fed a Western diet.

## 2 Experimental Section

### 2.1 Animals and Experimental Model

This study was conducted in accordance with the ARRIVE guidelines to ensure high standards in animal research reporting [26]. The experiments followed the *Guide for the Care and Use of Laboratory Animals* and were approved by the Ethics Committee on Animal Experiments of the Botucatu Medical School, São Paulo State University, UNESP (1337/2019 – CEUA). Male Wistar rats (± 187 g), approximately 40 days old, obtained from the Central Vivarium of Botucatu Medical School (São Paulo, Brazil), were used in the study. The animals were maintained under controlled environmental conditions: temperature (22°C ± 3°C), humidity (60% ± 5%), and a 12:12 h light–dark cycle with environmental enrichment. Throughout the experimental period, the rats were housed individually in boxes containing wood shavings to allow precise monitoring of individual food and water intake.

The animals were randomly distributed into four groups of seven animals each to receive: Control diet (C), control + PBJ (C+PBJ), WD, and WD + PBJ for 20 weeks. The WD groups also received 25% sucrose in their drinking water. The dietary model followed the protocol previously established and published by our research group and is presented in Table 1.

**TABLE 1:** Nutritional values of the diets.

Nutritional values	Control	Western diet
Protein (% of ingredients)	20.0	18.0
Carbohydrate (% of ingredients)	60.0	53.5
Fat (% of ingredients)	4.00	16.5
% Of unsaturated	69.0	47.0
% Of saturated	31.0	53.0
% Energy from protein	22.9	16.6
% Energy from carbohydrate	66.8	49.2
% Energy from fat	10.4	34.2
Energy (kcal/g)	3.59	4.35

*Note*: Western diet animals drink water with 25% sucrose.

After a 8‐h fasting period, the animals were anesthetized intraperitoneally with 120 mg/kg of thiopental [27, 28]. After confirming the absence of palpebral, foot, interdigital, and caudal reflexes, the animals were decapitated. Blood samples were collected in Falcon tubes containing anticoagulant and centrifuged (800 rpm;10 min; Eppendorf Centrifuge 5804‐R, Hamburg, Germany). Plasma and kidney tissue were then aliquoted and stored for subsequent analyses performed in duplicate. The nutritional profile was assessed according to the following parameters: caloric intake, body weight, and adiposity index. Caloric intake was determined by multiplying the energy value of each diet (g × kcal) by daily food consumption, plus the calories from the 25% sucrose solution provided to the WD groups.

### 2.2 Powder Bergamot Juice—Dosage Information/Regimen

The PBJ was kindly provided by the University of Milan (Italy). The preparation of the by‐product was carried out according to the technique described by Vedova et al. [17]. Briefly, Bergamot (*Citrus Bergamia* Risso & Poiteau) fruits (100 kg) from the three cultivars “Castagnaro”, “Femminello”, and “Fantastico” (equally represented), grown in the coastal area of Calabria (southern Italy), from Reggio Calabria to Monasterace, were harvested from November to February. The fruits were washed, peeled, and pressed, producing juice and a solid residue (pulp). The pulp was backwashed three times with deionized water and subjected to a pulping process using a decanter and centrifuge to obtain a technical juice, which was subsequently added to the fruit juice. Liquid–solid phase separation was performed using a rotary filter. The combined juices were then mixed with gum arabic (20%) to facilitate drying, reduce particle adhesion, and increase powder stability during storage [17]. Spray drying was performed in a semi‐industrial dryer, yielding in 2.8 kg of PBJ powder.

The animals received the PBJ at a dose of 250 mg/kg/day by gavage. Using body surface area conversion factor for interspecies dose extrapolation, this corresponds to approximately 2.43 g for a 60 kg human [29].

### 2.3 Nutritional Profile

Body weight was measured weekly. The adiposity index was used as an indicator of obesity, as provides a precise estimate of body fat accumulation. After euthanasia, the epididymal, visceral, and retroperitoneal fat depots were dissected to calculate the adiposity index using the formula: [(epididymal + retroperitoneal + visceral)/body weight x 100] [30].

### 2.4 Biochemical and Hormonal Parameters

Glycemia was assessed using an Accu‐Chek Performa glucometer (2014, Roche Diagnostics). Plasma was used to determine triacylglycerol levels (CELM kits, Barueri, São Paulo, Brazil). The method employed was enzymatic–colorimetric, and the analyses were performed using an automated device (Technicon, RA‐XTTM System, Global Medical Instrumentation, Minnesota, USA). Plasma insulin concentration was measured using a commercial ELISA kit (Elabscience Biotechnology Co., Ltd, USA, #E‐EL‐R3034), and absorbance was read with an Epoch microplate spectrophotometer (Agilent Technologies, Santa Clara, CA, USA; Serial No. 23021709).

Homeostasis Model Assessment of Insulin Resistance (HOMA‐IR) and the triglyceride–glucose (TyG) index were used as markers of insulin resistance. They were calculated using the following formulas: HOMA‐IR = (fasting glucose (mmol/L) × fasting insulin (µU/mL)/22.5)) and TyG = Ln [fasting triglycerides (mg/dL) × fasting glucose (mg/dL)]/2 [31, 32].

### 2.5 Systolic Blood Pressure (SBP)

SBP analysis was performed using the tail–cuff plethysmography technique according to Pfeffer et al. [33], using a Narco Bio‐System PE 300 electrosphygmomanometer, model 709‐0610 (International Biomedical, Inc, Houston, TX, USA) [34]. This method does not allow diastolic blood pressure to be assessed. The animals were heated (40°C) for 5 min in a wooden box (50 × 40 cm) lined with autoclaved pine shavings to promote vasodilation of the caudal artery. The cuff connected to the pulse transducer was coupled to the animal's tail and was subsequently inflated (200 mmHg) and deflated to record arterial pulsations. Measurements were collected from a Gould RS 3200 polygraph (Gould Instrument Valley View, Ohio, USA).

### 2.6 Renal Function

Urine was collected 4 days before euthanasia over a 12‐h period using individual metabolic cages. Creatinine and total protein concentrations were measured using commercial colorimetric kits (CELM kits, Barueri, São Paulo, Brazil). To assess renal function, proteinuria and glomerular filtration rate (GFR) were evaluated [35].

### 2.7 Oxidative Stress Parameters in Kidneys

The effect of PBJ on the kidneys was assessed by measuring levels of malondialdehyde (MDA), 4‐hydroxynonenal (4‐HNE), advanced oxidation proteins products (AOPP), protein carbonylation (CBO), and the activity of the antioxidant enzymes superoxide dismutase (SOD), and catalase (CAT), as follows:

#### 2.7.1 Sample Preparation

Tissues (∼100 mg) were homogenized with ice‐cold phosphate‐buffered saline (PBS), 1 mL, pH 7.4) for oxidative damage assays or 1.15 % KCl solution for enzymatic activity assays (SOD and CAT) using the ULTRA‐TURRAX T25 basic IKA Werke Staufen/Germany. The homogenates were centrifuged at 3500 rpm for 10 min at 4°C. A Spectra Max 190 microplate spectrophotometer (Molecular Devices, Sunnyvale, CA, USA) was used for the readings [36].

#### 2.7.2 MDA

To assess MDA levels (nmol/mg of protein), thiobarbituric acid (TBA, 0.67%) was added to the supernatant (2:5; TBA: Supernatant), followed by centrifugation at 3500 rpm for 10 min. The samples were then heated for 45 min in a water bath and, after cooling, transferred to a 96‐well plate for reading at 532 and 600 nm. MDA concentration was calculated using the molar extinction coefficient (1.56 × 105 M^−1^ cm^−1^) [36, 37].

#### 2.7.3 CBO

CBO was assessed using the 2,4‐dinitrophenylhydrazine (DNPH) method [38]. A total of 100 µL of sample was mixed with 100 µL of DNPH (10 nM) and incubated for 10 min. The DNPH‐treated wells were then incubated with 50 µL of sodium hydroxide (NaOH, 6 M) for an additional 10 min. Absorbance was read at 450 nm, and the results were calculated with a molar extinction coefficient of DNPH (22,000 M^−1^ cm^−1^) and expressed as nmol/mg protein [39].

#### 2.7.4 AOPP

AOPP levels were determined according to Kalousová et al. [40]. Tissue supernatant samples (200 µL) were diluted 1:5 in PBS. Chloramine T (0‐100 µmol/L; 200 µL) was used for the calibration curve, and PBS (200 µL) served as the blank. Samples were placed into a microplate and mixed with 10 µL of 1.16 M potassium chloride solution (KCl) and 20 µL of acetic acid. Absorbance was measured immediately at 340 nm, and AOPP levels were expressed as chloramine units (µmol/L).

#### 2.7.5 4‐HNE

The 4‐HNE levels were quantified using a commercial ELISA kit (Elabscience Biotechnology Co., Ltd, USA, E‐EL‐0128), following the manufacturer's instructions.

#### 2.7.6 Activity of the Enzymes SOD and CAT

SOD activity was determined according to CROUCH et al. [41], based on the enzyme's ability to inhibit the reduction of nitroblue tetrazolium (NBT) by free radicals generated by hydroxylamine in an alkaline medium (pH 10). Hydroxylamine generates an O_2_^−^ flux that reduces NBT to blue‐formazan at room temperature. With the addition of the sample, the rate of NBT reduction decreases proportionally to SOD activity. Results were expressed as U/mg total protein. CAT activity was determined in phosphate buffer (pH 7.0) using 0.5 mL of sample and 30% hydrogen peroxide. Absorbance was recorded at 240 nm [42].

### 2.8 Statistical Analysis

Data distribution were assessed using the Shapiro–Wilk test (*p* > 0.05). Variables that followed a parametric distribution were expressed as mean ± standard deviation, whereas non‐parametric variables were presented as median (interquartile range). For normally distributed data, group comparisons were performed using two‐way ANOVA followed by Tukey's post‐hoc test. For non‐parametric data, the Kruskal–Wallis test was applied, followed by Dunn's post‐hoc test when appropriate. Pearson's correlation analysis was applied to examine associations between metabolic parameters, OS markers, and renal function variables. Additionally, linear regression analyses were conducted to further explore individual relationships between selected variables. Statistical analyses were carried out using SigmaPlot 15.0, and graphs were created using GraphPad Prism Version 10.1.2. Statistical significance was set at *p* < 0.05.

The sample size calculation for the experimental design was based on the expected outcomes. A significance level of *α* = 5% and a power of 1–*β* = 80% (OpenEpi 3.01) were considered. The chosen 95% confidence interval ensured that the observations accurately represented the population studied, thereby reducing the chance of type I error. A 1:1 sample ratio between groups was adopted to ensure numerical equivalence and account for potential experimental losses, resulting in an adequate number of animals to support robust statistical comparisons [43, 44].

## 3 Results

### 3.1 Nutritional, Biochemical, and Hormonal Profiles

The nutritional, metabolic, and hormonal profiles of the animals are presented in Table 2. Compared to the C group, the WD group showed higher levels of total caloric intake, weight gain, final body weight, adiposity index, glucose, triglycerides, SBP, and TyG, respectively (F (1,24) = 25.862, *p* < 0.001; F (1,24) = 22.255, *p* < 0.001; F (1,24) = 20.619, *p* < 0.001; F (1,24) = 118.424, *p* < 0.001; F (1,24) = 14.994, *p* = 0.013; F (1, 24) = 100.100, *p* < 0.001; F (1,24) = 32.997, *p* < 0.001; H (3) = 12.270, *p* = 0.010. Tukey's and Dunn's post hoc test revealed that the WD group had significantly higher values compared to the Control (all p < 0.001; except glucose and TyG; *p* = 0.013 and *p* = 0.015), Control + PBJ, and WD + PBJ groups (*p* = 0.009; *p* = 0.023; *p* = 0.037; *p* < 0.001; *p* = 0.010; *p* < 0.001; *p* = 0.006; *p* = 0.712). Similarly, the WD + PBJ group exhibited increases in the same parameters relative to the C + PBJ group.

**TABLE 2:** Nutritional, metabolic, and hormonal analysis in the 20th week.

	Groups			
Variables	C	C + PBJ	WD	WD + PBJ
Caloric Intake (kcal/day)	64.08 ± 4.90	62.10 ± 5.33	78.91 ± 8.06^a^	71.86 ± 6.77^b^
Initial Body Weight (g)	182.39 ± 21.00	189.14 ± 17.86	194.97 ± 13.69	191.10 ± 18.35
Final Body Weight (g)	460.66± 48.50	470.23 ± 75.66	572.99 ± 20.00^a^	529.40 ± 38.84^b^
Body Weight Gain (g)	278.27 ± 43.92	281.09 ± 66.59	378.01 ± 24.92^a^	338.31 ± 27.67^b^
Adiposity Index (%)	2.14 ± 0.33	3.17 ± 1.58	8.14 ± 1.40^a^	6.68 ± 0.89^b, c^
Glucose (mg/dL)	79.86 ± 9.74	79.43 ± 6.83	90.71 ± 6.24^a^	90.71 ± 6.97^b^
Triglycerides (mg/dL)	22.57 ± 6.78	28.00 ± 5.16	98.14 ± 25.81^a^	61.29 ± 9.48^b, c^
Insulin (µU/mL)	4.60 (3.00 ‐ 31.20)	3.30 (3.10 ‐ 5.10)	3.90 (3.10 ‐ 4.90)	3.40 (3.20 ‐ 8.10)
HOMA‐IR	0.60 (0.70 ‐ 1.10)	0.70 (0.60 ‐ 1.10)	0.90 (0.70 ‐ 1.10)	0.70 (0.60 ‐ 1.10)
TyG	6.84 (6.32 ‐ 7.09)	7.01 (4.51 ‐ 7.23)	8.41 (5.29 ‐ 8.81) ^a^	7.91 (5.83 ‐ 8.32) ^b^
Systolic Blood Pressure (mmHg)	120.95 ± 6.20	119.76 ± 7.25	145.48 ± 8.82^a^	134.57 ± 12.62^b, c^

Data are expressed as mean ± standard deviation and median with interquartile range (*n* = 7/group). Normality was assessed using the Shapiro–Wilk test (*p* > 0.05). For parametric data, a comparison by two‐way ANOVA with Tukey post‐hoc was made. For nonparametric data, the comparison was performed by Kruskal–Wallis with Dunn's post‐hoc, when there was a significant difference between the groups. Note: The premise of homoscedasticity was verified and considered met (*p* > 0.05). a‐ *p* < 0.05 vs C; b‐ *p* < 0.05 vs C+PBJ; c‐ *p* < 0.05 vs WD. C—Control group; PBJ — Powder Bergamot Juice; WD —Western Diet.

On the other hand, PBJ supplementation reduced the adiposity index, triglycerides, and SBP levels compared to the WD group (*p* = 0.027; *p* < 0.001; *p* = 0.034).

### 3.2 Renal Function Parameters

**FIGURE 1:**
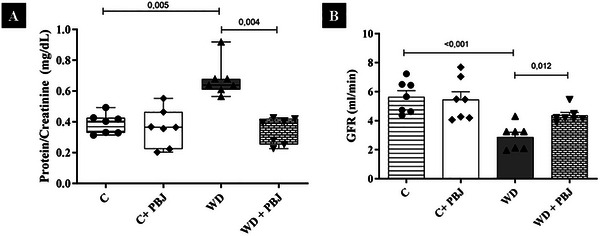
Renal function in 20 weeks (*n* = 7/group). (A) Protein/Creatinine ratio (mg/dL); (B) Glomerular Filtration Rate—GFR (mL/min). C—Control group; PBJ—Powder Bergamot Juice; WD — Western Diet. Data are expressed as mean ± standard deviation and median with interquartile range. Data normality was checked by Shapiro–Wilk test (*p* > 0.05). Comparison by two‐way ANOVA with Tukey post‐hoc. For non‐parametric data, the comparison was performed by Kruskal–Wallis, with Dunn's post‐hoc. The premise of homoscedasticity was verified and considered met (*p* > 0.05).

Renal function parameters are presented in Figure 1. The WD group showed higher protein/creatinine and lower GFR compared to the C group, respectively (*H* (3) = 15.570, *p* = 0.026; *F* (1,24) = 24.172, *p* < 0.001). Notably, the addition of PBJ (WD + PBJ) resulted in lower protein/creatinine levels and higher GFR compared to the WD group (*p* = 0.004; *p* = 0.012).

### 3.3 Oxidative Stress Markers in Kidneys

**FIGURE 2:**
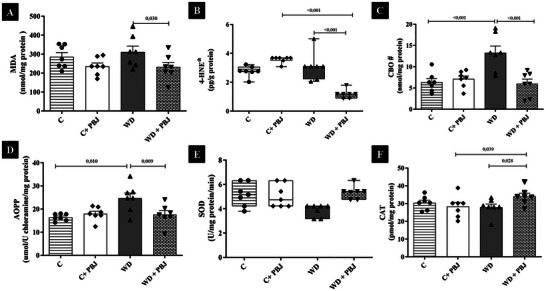
Oxidative Stress in 20 weeks (*n* = 7/group). (A) Malondialdehyde—MDA (nmol/ mg of protein); (B) 4‐hydroxynonenal – 4‐HNE (pg/ g of protein); (C) Protein carbonylation—CBO (nmol/ mg protein); (D) Products of advanced oxidation of proteins—AOPP (umol/ U chloramine/ mg protein); (E) Superoxide dismutase (U/ mg protein/ min); (F) Catalase (pmol/ mg protein). C—Control group. PBJ—Powder Bergamot Juice. WD — Western Diet. Data are expressed as mean ± standard deviation and median with interquartile range. Data normality was checked by Shapiro–Wilk test, *p* > 0.05. Comparison by two‐way ANOVA with Tukey post‐hoc, *p* < 0.05. For non‐parametric data, the comparison was made by Kruskal–Wallis, with Dunn's post‐hoc. #; *—The analysis assumes homoscedasticity, confirmed by the Equal Variance Test (*p* < 0.05).

The OS parameters are shown in Figure 2. The WD group exhibited higher CBO and AOPP levels compared to the C group (F (1,24) = 6.634, *p* < 0.001; F (1,24) = 3.512, *p* = 0.010). On the other hand, PBJ supplementation reduced MDA (F (1,24) = 0.184, *p* = 0.030), 4‐HNE (H (3) = 20.54, *p* = 0.014), CBO (*p* < 0.001), and AOPP levels (*p* = 0.009), and increased CAT (F (1,24) = 0.743, *p* = 0.028) relative to the WD group. PBJ also decreased 4‐HNE levels and improved CAT activity compared to the C+ PBJ group (*p* < 0.001 and *p* = 0.039). In addition, the C + PBJ group showed increased 4‐HNE levels compared to the C group (*p* = 0.145).

### 3.4 Association Between Renal Impairment and Pathophysiological Factors

**FIGURE 3:**
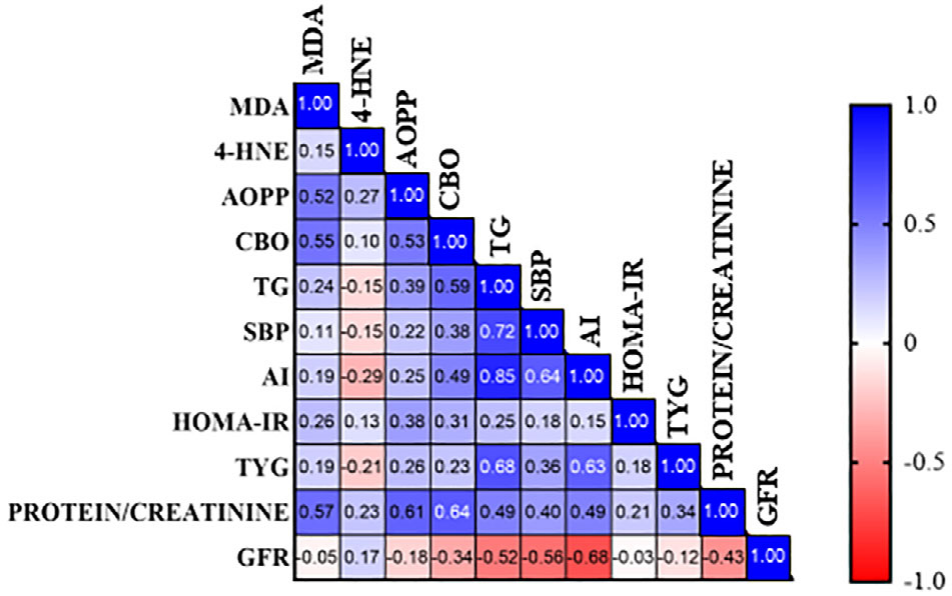
Correlation matrix among variables (*n* = 7/ group). Positive correlations are represented in blue and negative correlations are represented in red. The intensity of the color is proportional to the correlation coefficients. MDA—Malondialdehyde; 4‐HNE – 4‐Hydroxynonenal; AOPP‐ Advanced Protein Oxidation Products; CBO—Protein Carbonylation; TG—Triglycerides; SBP—Systolic Blood Pressure; AI—Adiposity Index; HOMA‐IR—Homeostasis Model Assessment of Insulin Resistance; TyG—Triglycerides‐glucose index; GFR—Glomerular Filtration Rate. Statistical analysis was performed using Pearson's correlation.

Figure 3 shows the Pearson correlation analysis between plasma metabolic parameters, renal OS, and function. The adiposity index, triglycerides, and SBP were significantly and moderately correlated with both protein/creatinine and GFR, whereas AOPP, CBO, and MDA correlated significantly only with GFR.

**FIGURE 4:**
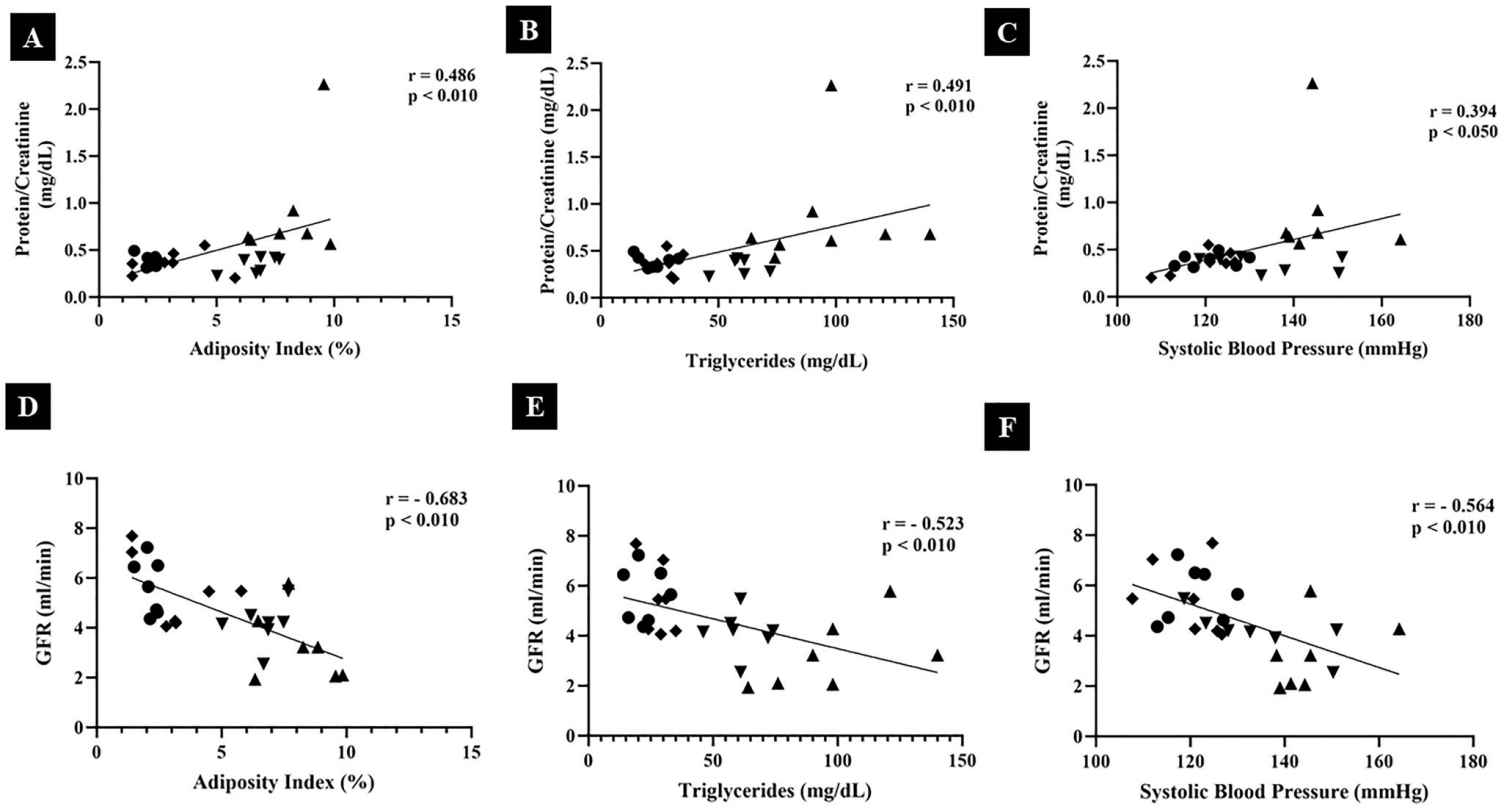
Linear regression among metabolic parameters in relation to Renal Function (*n* = 7/ group). (A) Adiposity Index (%)/ Protein/Creatinine (mg/ dL); (B) Triglycerides (mg/ dL)/Protein/Creatinine (mg/ dL); (C) Systolic Blood Pressure (mmHg)/Protein/Creatinine (mg/ dL); (D) Adiposity Index (%)/GFR‐ Glomerular Filtration Rate (ml/ min); (E) Triglycerides (mg/ dL)/GFR‐ Glomerular Filtration Rate (ml/ min); (F) Systolic Blood Pressure (mmHg)/GFR‐ Glomerular Filtration Rate (ml/ min). Linear regression was used to examine the association between the variables. Filled circles represent Control Group; diamonds represent the Control + PBJ Group; upward‐pointing triangles represent the Western Diet Group; downward‐pointing triangles represent the Western Diet + PBJ Group.

Figure 4 illustrates the individual linear regression analyses for the metabolic parameters correlated with both protein/creatinine ratio and GFR, namely adiposity index, triglycerides, and SBP. Regarding protein/creatinine, the adiposity index (F (1,26) = 8.079, *p* = 0.0086, *r* = 0.486), SBP (F (1,26) = 4.807, *p* = 0.0375, *r* = 0.394), and triglycerides (F (1,26) = 8.283, *p* = 0.0079, *r* = 0.491) were all significant predictors. Similarly, for GFR, the adiposity index (F (1.26) = 22.83, *p* < 0.0001, *r* = 0.683), SBP (F (1.26) = 12.16, *p* = 0.0018, *r* = 0.564) and triglycerides (F (1.26) = 9.800, *p* = 0.0043, *r* = 0.523) were also significantly associated.

Table 3 shows the regression performed in a stratified manner for each of the groups. A significant effect was observed only in the WD + PBJ group, in which higher AI and SBP were associated with lower GFR (*p* = 0.045).

**TABLE 3:** Stratified regression of renal and pathophysiological parameters.

Treatment	β	ep	CI95%		*p*
**Protein/Creatinine vs. AI**					
C	−0.094	0.077	−0.292	0.103	*0.274*
C + PBJ	0.006	0.035	−0.084	0.095	*0.873*
WD	0.194	0.174	−0.254	0.642	*0.317*
WD + PBJ	0.062	0.033	−0.024	0.148	*0.122*
					
**Protein/Creatinine vs. Triglycerides**					
C	−0.002	0.004	−0.013	0.009	*0.633*
C + PBJ	0.000	0.010	−0.027	0.027	*0.996*
WD	0.000	0.011	−0.027	0.028	*0.969*
WD + PBJ	0.003	0.004	−0.007	0.013	*0.443*
					
**Protein/Creatinine vs. PAS**					
C	0.003	0.005	−0.009	0.015	*0.554*
C + PBJ	0.012	0.005	−0.002	0.026	*0.077*
WD	−0.005	0.031	−0.084	0.074	*0.875*
WD + PBJ	−0.002	0.003	−0.010	0.005	*0.465*
					
**GFR vs. AI**					
C	0.022	0.080	−0.182	0.227	*0.791*
C + PBJ	−0.064	0.084	−0.280	0.152	*0.482*
WD	0.073	0.064	−0.090	0.237	*0.301*
**WD + PBJ**	−**0.053**	**0.020**	−**0.104**	−**0.002**	***0.045***
					
**GFR vs. Triglycerides**					
C	0.000	0.073	−0.189	0.188	*0.997*
C + PBJ	−0.140	0.108	−0.417	0.137	*0.252*
WD	0.032	0.020	−0.019	0.083	*0.169*
WD + PBJ	−0.006	0.041	−0.111	0.099	*0.883*
					
**GFR vs. PAS**					
C	0.022	0.080	−0.182	0.227	*0.791*
C + PBJ	−0.064	0.084	−0.280	0.152	*0.482*
WD	0.073	0.064	−0.090	0.237	*0.301*
**WD + PBJ**	**—0.053**	**0.020**	−**0.104**	−0**.002**	***0.045***

*Note*: β—Regression coefficient; ep—Standard error; CI95%—Confidence Interval 95%; AI—Adiposity Index (%); GFR (mL/min), Triglycerides (mg/dL); SBP—Systolic Blood Pressure (mmHg). Significance level: *p* <0.05. C—Control group; PBJ — Powder Bergamot Juice; WD —Western Diet.

## 4 Discussion

The WD is characterized by the intake of energy‐dense, nutrient‐poor foods such as fast foods, soft drinks, and ultraprocessed products rich in added sugars, salt, and saturated fats [45]. Chronic consumption of these foods is associated with several metabolic disturbances, including increased fat mass, dyslipidemia, insulin resistance, elevated blood pressure, and OS. These metabolic and hemodynamic alterations have been implicated in the pathogenesis of kidney disease [46].

Therapeutic strategies have therefore been investigated to mitigate the harmful effects of WD. Polyphenols have been widely investigated due to their antioxidant properties, acting as free radical scavengers, and are also recognized for their ability to modulate cell signaling pathways involved in metabolic regulation, independently of reactive oxygen species (ROS) production [47, 48, 49]. Therefore, the aim of this study was to evaluate the potential preventive effect of PBJ on renal pathophysiological processes in Wistar rats fed a WD, as well as its impact on associated factors such as OS, dyslipidemia, and body fat accumulation.

The diet used in this experiment was intended to mimic the WD pattern commonly consumed by humans, and it was associated with relevant changes such as proteinuria, decreased GFR, OS in the kidneys, increased blood pressure, body fat and dyslipidemia. These data are consistent with the literature, which reports the association of such alterations with the consumption of a WD [50, 51]. Studies conducted with bergamot have shown beneficial associations of fruit and leaf extracts on metabolic alterations and blood pressure levels [52]. Our results are relevant because these variations are among the pathophysiological mechanisms that are known to contribute to dysfunction in many organs, including the kidneys [53].

The biological effects of phytochemicals largely depend on both the individual and total secondary metabolite content, as well as on geographical origin, environmental conditions, and genetic factors. Evidence shows that phytochemical composition can vary substantially with soil type, climate, harvest dates, altitude, and plant growth stage, ultimately influencing their potential biological activity [54]. In this study, PBJ was consistent with most of these aspects, except for the date of harvest, which may represent a limitation, since different seasons can influence the bioavailability of bioactive compounds.

The intake of bioactive compounds has been associated with the prevention of redox imbalance and with a reduction in OS‐induced injury. Some evidence indicates that flavonoids present in bergamot are associated with reno‐protective effects. Gelen et al. demonstrated that naringin alleviates oxidative DNA damage and nephrotoxicity by reducing 8‐hydroxy‐2'‐deoxyguanosine (8‐OHdG) expression [55]. Similarly, naringin has been shown to counteract nephrotoxicity induced by 5‐fluorouracil by modulating OS, apoptosis, autophagy, and inflammation [56]. These investigations are consistent with our results, as PBJ is rich in naringenin; thus, this flavonoid may have contributed to the reduction in MDA and 4‐HNE, which is consistent with the preservation of renal function and with a mitigation of kidney injury in the WD + PBJ group compared to the WD group.

Interestingly, the WD alone did not significantly elevate these markers compared with controls. Nevertheless, PBJ group supplementation was associated with a significant reduction in MDA and 4‐HNE levels, particularly in animals exposed to the WD diet. These findings suggest that PBJ can be especially effective under conditions of metabolic insult, as reductions in OS biomarkers are often associated with organ‐level benefits. Another intriguing finding was that the C + PBJ group showed higher 4‐HNE levels compared to the WD + PBJ group. Since no differences were observed between C and C + PBJ for this parameter, these results were unexpected. Because lipid peroxidation was not a predominant feature in this model, further investigation is required to better interpret these observations. Importantly, this finding did not translate into functional renal impairment and may reflect context‐dependent lipid peroxidation dynamics.

Regarding protein oxidation, renal protein damage was assessed through AOPP and CBO. AOPP levels reflect protein oxidation caused by free radicals, whereas CBO represents irreversible oxidative modifications that often impair protein function and serve as indicators of severe oxidative damage and disease‐related protein dysfunction. These carbonyl groups result from interactions between proteins and oxidation products of lipids, sugars, and proteins [57]. Both CBO and AOPP were increased in the WD but were attenuated by PBJ supplementation. These experiments indicate that the WD diet may have favored greater protein oxidation with consequent protein carbonylation, which is particularly relevant since CBO represents irreversible damage, unlike AOPP, which may still be partially repaired by endogenous mechanisms. The accumulation of CBO has been associated with permanent structural modifications, loss of protein function, and progression of cellular dysfunction and disease [58, 59, 60]. Conversely, PBJ group was associated with the prevention of increases in both AOPP and CBO, underscoring its role in attenuating long‐term oxidative damage and in preserving protein integrity under conditions of increased OS [58].

In this study, the antioxidant enzymes activity was also assessed. CAT activity was increased in the WD + PBJ group, aligning with previous research demonstrating the antioxidant actions of citrus‐derived bioactive compounds [10, 61, 62, 63]. Collectively, these data support the role of dietary polyphenols in modulating OS, particularly in conditions associated with increased protein oxidation [58]. Moreover, attenuation of triglyceride levels, together with reduced fat deposition may have contributed to decreased proteinuria and to improved GFR [64].

Regarding the action of PBJ on other pathophysiological mechanisms, favorable associations were observed. A reduction in SBP levels in the WD + PBJ group relative to the WD group was observed. Several studies indicate that natural polyphenols may play an important role in preventing vascular alterations [65, 66, 67, 68]. For instance, in patients with CKD, dietary nitrate supplementation by administration of beetroot juice reduced SBP and the renal resistance index, which may potentially reduce the development of cardiovascular and renal diseases [69]. In addition, natural polyphenols have been reported to prevent endothelial dysfunction under conditions of impaired renal vascularity through mechanisms involving antioxidant activity, possible activation of reactive nitrogen species, and inhibition of metalloproteinases [70]. In hypertensive rats, quercetin, one of the flavonoids present in PBJ, has been shown to improve endothelium‐dependent aortic dilation [61]. Da Costa et al. also showed that polyphenols derived from *Euterpe oleracea* Mart. are associated with the prevention of endothelial dysfunction and vascular structural changes in hypertensive rats, decreasing MDA and CBO levels and restoring endogenous antioxidant enzyme activities [71].

Regarding dyslipidemia, plasma triglyceride levels were lower in the WD + PBJ, which may be attributable to bergamot. The hypolipidemic effects of *Citrus* species have been attributed to several components, such as flavonoids, pectins, and ascorbic acid. Flavonoids have been proposed to inhibit LDL oxidation, increase LDL receptor‐mediated uptake, and enhance fecal excretion of bile acids [72, 73]. In addition, fecal output of total bile acids and neutral sterols has been shown to be higher in bergamot‐treated hyperlipidemic animals [74]. Another study demonstrated that pectins and flavonoids lower serum cholesterol levels by modulating hepatic HMG‐CoA concentrations [75]. Altogether, these findings suggest that bergamot acts through multiple mechanisms to control dyslipidemia, which is consistent with the improvements observed in the present study.

Nonetheless, attenuation of fat accumulation in the WD + PBJ group may also have contributed to preventing kidney damage. Visceral adipose tissue has been strongly associated with adverse cardiometabolic parameters and with declines in kidney function [76, 77]. However, the increase in adiposity is accompanied by other comorbidities, including hypertension, dyslipidemia, and OS, arising from adipocyte hypertrophy. Thus, the association between increased adiposity and kidney disease should not be considered an isolated factor. In the present study, reductions in hypertension, dyslipidemia, OS, and renal dysfunction were observed in parallel with the prevention of increased adiposity index in PBJ‐treated animals.

Plant‐derived bioactive compounds have been reported to be effective in combating obesity by modulating several mechanisms, including inhibition of pancreatic lipase and glucosidase, appetite suppression, stimulation of thermogenesis and lipid metabolism, and inhibition of adipogenesis and fat accumulation [78]. Specifically with bergamot, Lo Furno et al. demonstrated that *Citrus bergamia* extract is associated with reduced adipogenesis and with increased lipolysis by modulating peroxisome proliferator‐activated receptor gamma (PPARγ) levels in mesenchymal stem cells from human adipose tissue [79]. Together, these studies highlight the anti‐obesity potential of bergamot bio‐actives, which is consistent with the anti‐adipogenic effects observed with PBJ in this study.

Finally, considering all these results, linear regression analyses were conducted to determine which pathophysiological factors were most strongly associated with renal dysfunction in this experimental model. These analyses demonstrated that metabolic parameters, particularly adiposity index, triglycerides, and SBP, exhibited stronger and more consistent associations with renal function indicators than OS markers. These findings suggest that metabolic dysregulation plays a predominant role in renal impairment within this model. Although OS markers such as AOPP, CBO, and MDA showed significant associations with GFR, their correlations were more limited compared to those observed in metabolic variables. These results align with clinical studies identifying visceral adiposity and hypertriglyceridemia as key predictors of CKD [80, 81], as well as evidence linking hypertension to renal damage through sympathetic overactivity and vascular dysfunction [82, 83]. In parallel, when the data were analyzed in a stratified manner, relating to GFR and protein/creatinine ratio with pathophysiological factors (AI, triglycerides, and SBP), it was observed that in the WD + PBJ group, higher AI and SBP levels were associated with lower GFR (Table 3).

Notably, the WD + PBJ group is positioned between the WD and control groups, suggesting that PBJ supplementation was associated with a partial but meaningful modulatory effect. Overall, these discoveries highlight the central role of metabolic alterations in kidney injury and support the potential contributory role of PBJ in attenuating both metabolic and renal disturbances, particularly under the influence of high sugar‐fat diets.

In summary, our results suggest reno‐protective effects of PBJ against pathophysiological parameters associated with kidney disease, by the combined modulation of metabolic parameters and redox balance.

This study is a preclinical experiment with limited sample size; therefore, further studies involving both animal models and human translational research are required to investigate factors such as dose‐dependence, bioavailability, and interspecies differences. The insights gained from this study may serve as a valuable reference for researchers to validate these results to further explore the therapeutic potential of PBJ in preventing metabolic alterations and elevated blood pressure associated with WD. In addition, future studies are necessary to elucidate the effects of specific bioactive compounds on renal dysfunction in diet‐induced obesity models.

## 5 Conclusion

PBJ attenuated crucial metabolic and oxidative alterations induced by a WD, highlighting its potential as a preventive strategy against early mechanisms associated with CKD.

## Funding

This work was supported by the Coordination for the Improvement of Higher Education Personnel (CAPES—Brazil), under grant (88887.899588/2023‐00); the São Paulo Research Foundation (FAPESP) for funding the project under grant (2021/13050‐2), and the National Council for Scientific and Technological Development (CNPq—Brazil) (306640/2023‐6).

## Ethics Statement

This study was conducted in accordance with the ARRIVE guidelines to ensure high standards in the reporting of animal research. The experiments followed the Guide for the Care and Use of Laboratory Animals and approved by the Ethics Committee on Animal Experiments of the Botucatu Medical School, São Paulo State University, UNESP (1337/2019 ‐CEUA).

## Declaration of Consent to Participation

Not applicable.

## Conflicts of Interest

The authors declare no conflicts of interest.

## Data Availability

The data that support the findings of this study are available from the corresponding author upon reasonable request.
